# Moving from Data on Deaths to Public Health Policy in Agincourt, South Africa: Approaches to Analysing and Understanding Verbal Autopsy Findings

**DOI:** 10.1371/journal.pmed.1000325

**Published:** 2010-08-17

**Authors:** Peter Byass, Kathleen Kahn, Edward Fottrell, Mark A. Collinson, Stephen M. Tollman

**Affiliations:** 1 Umeå Centre for Global Health Research, Department of Public Health and Clinical Medicine, Umeå University, Sweden; 2 MRC/Wits Rural Public Health and Health Transitions Research Unit (Agincourt), School of Public Health, Faculty of Health Sciences, University of the Witwatersrand, Johannesburg, South Africa; World Health Organization, Switzerland

## Abstract

**Background:**

Cause of death data are an essential source for public health planning, but their availability and quality are lacking in many parts of the world. Interviewing family and friends after a death has occurred (a procedure known as verbal autopsy) provides a source of data where deaths otherwise go unregistered; but sound methods for interpreting and analysing the ensuing data are essential. Two main approaches are commonly used: either physicians review individual interview material to arrive at probable cause of death, or probabilistic models process the data into likely cause(s). Here we compare and contrast these approaches as applied to a series of 6,153 deaths which occurred in a rural South African population from 1992 to 2005. We do not attempt to validate either approach in absolute terms.

**Methods and Findings:**

The InterVA probabilistic model was applied to a series of 6,153 deaths which had previously been reviewed by physicians. Physicians used a total of 250 cause-of-death codes, many of which occurred very rarely, while the model used 33. Cause-specific mortality fractions, overall and for population subgroups, were derived from the model's output, and the physician causes coded into comparable categories. The ten highest-ranking causes accounted for 83% and 88% of all deaths by physician interpretation and probabilistic modelling respectively, and eight of the highest ten causes were common to both approaches. Top-ranking causes of death were classified by population subgroup and period, as done previously for the physician-interpreted material. Uncertainty around the cause(s) of individual deaths was recognised as an important concept that should be reflected in overall analyses. One notably discrepant group involved pulmonary tuberculosis as a cause of death in adults aged over 65, and these cases are discussed in more detail, but the group only accounted for 3.5% of overall deaths.

**Conclusions:**

There were no differences between physician interpretation and probabilistic modelling that might have led to substantially different public health policy conclusions at the population level. Physician interpretation was more nuanced than the model, for example in identifying cancers at particular sites, but did not capture the uncertainty associated with individual cases. Probabilistic modelling was substantially cheaper and faster, and completely internally consistent. Both approaches characterised the rise of HIV-related mortality in this population during the period observed, and reached similar findings on other major causes of mortality. For many purposes probabilistic modelling appears to be the best available means of moving from data on deaths to public health actions.

*Please see later in the article for the Editors' Summary*

## Abstract 2

Peter Byass and colleagues compared two methods of assessing data from verbal autopsies, review by physicians or probabilistic modeling, and show that probabilistic modeling is the most efficient means of analyzing these data

## Editors' Summary

**Background:**

Whenever someone dies in a developed country, the cause of death is determined by a doctor and entered into a “vital registration system,” a record of all the births and deaths in that country. Public-health officials and medical professionals use this detailed and complete information about causes of death to develop public-health programs and to monitor how these programs affect the nation's health. Unfortunately, in many developing countries dying people are not attended by doctors and vital registration systems are incomplete. In most African countries, for example, less than one-quarter of deaths are recorded in vital registration systems. One increasingly important way to improve knowledge about the patterns of death in developing countries is “verbal autopsy” (VA). Using a standard form, trained personnel ask relatives and caregivers about the symptoms that the deceased had before his/her death and about the circumstances surrounding the death. Physicians then review these forms and assign a specific cause of death from a shortened version of the International Classification of Diseases, a list of codes for hundreds of diseases.

**Why Was This Study Done?:**

Physician review of VA forms is time-consuming and expensive. Consequently, computer-based, “probabilistic” models have been developed that process the VA data and provide a likely cause of death. These models are faster and cheaper than physician review of VAs and, because they do not rely on the views of local doctors about the likely causes of death, they are more internally consistent. But are physician review and probabilistic models equally sound ways of interpreting VA data? In this study, the researchers compare and contrast the interpretation of VA data by physician review and by a probabilistic model called the InterVA model by applying these two approaches to the deaths that occurred in Agincourt, a rural region of northeast South Africa, between 1992 and 2005. The Agincourt health and sociodemographic surveillance system is a member of the INDEPTH Network, a global network that is evaluating the health and demographic characteristics (for example, age, gender, and education) of populations in low- and middle-income countries over several years.

**What Did the Researchers Do and Find?:**

The researchers applied the InterVA probabilistic model to 6,153 deaths that had been previously reviewed by physicians. They grouped the 250 cause-of-death codes used by the physicians into categories comparable with the 33 cause-of-death codes used by the InterVA model and derived cause-specific mortality fractions (the proportions of the population dying from specific causes) for the whole population and for subgroups (for example, deaths in different age groups and deaths occurring over specific periods of time) from the output of both approaches. The ten highest-ranking causes of death accounted for 83% and 88% of all deaths by physician interpretation and by probabilistic modelling, respectively. Eight of the most frequent causes of death—HIV, tuberculosis, chronic heart conditions, diarrhea, pneumonia/sepsis, transport-related accidents, homicides, and indeterminate—were common to both interpretation methods. Both methods coded about a third of all deaths as indeterminate, often because of incomplete VA data. Generally, there was close agreement between the methods for the five principal causes of death for each age group and for each period of time, although one notable discrepancy was pulmonary (lung) tuberculosis, which accounted for 6.4% and 21.3% of deaths in this age group, respectively, according to the physicians and to the model. However, these deaths accounted for only 3.5% of all the deaths.

**What Do These Findings Mean?:**

These findings reveal no differences between the cause-specific mortality fractions determined from VA data by physician interpretation and by probabilistic modelling that might have led to substantially different public-health policy programmes being initiated in this population. Importantly, both approaches clearly chart the rise of HIV-related mortality in this South African population between 1992 and 2005 and reach similar findings on other major causes of mortality. The researchers note that, although preparing the amount of VA data considered here for entry into the probabilistic model took several days, the model itself runs very quickly and always gives consistent answers. Given these findings, the researchers conclude that in many settings probabilistic modeling represents the best means of moving from VA data to public-health actions.

**Additional Information:**

Please access these Web sites via the online version of this summary at http://dx.doi.org/10.1371/journal.pmed.1000325.

- The importance of accurate data on death is further discussed in a perspective previously published in *PLoS Medicine* Perspective by Colin Mathers and Ties Boerma
- The World Health Organization (WHO) provides information on the vital registration of deaths and on the International Classification of Diseases; the WHO Health Metrics Network is a global collaboration focused on improving sources of vital statistics; and the WHO Global Health Observatory brings together core health statistics for WHO member states
- The INDEPTH Network is a global collaboration that is collecting health statistics from developing countries; it provides more information about the Agincourt health and socio-demographic surveillance system and access to standard VA forms
- Information on the Agincourt health and sociodemographic surveillance system is available on the University of Witwatersrand Web site
- The InterVA Web site provides resources for interpreting verbal autopsy data and the Umeå Centre for Global Health Reseach, where the InterVA model was developed, is found at http://www.globalhealthresearch.net
- A recent *PLoS Medicine* Essay by Peter Byass, lead author of this study, discusses The Unequal World of Health Data

## Introduction

Throughout the history of public health, the concept of recording causes of individual deaths in a population and presenting them in aggregate form has been a central component of understanding health and disease at the community level. This continues to be the case, even though the extent and quality of cause of death data varies widely around the world [1].

For a large proportion of the world's communities in which individual deaths are not routinely recorded and classified by cause as part of routine civil and health service procedures, verbal autopsy (VA) has become an important technique [2]. VA involves interviewing family, friends, or carers after a death has occurred, to find out about the circumstances of death. These data are normally collected by lay interviewers, and their findings are later interpreted into possible cause(s) of death. Approaches to undertaking the interviews and interpreting the findings vary and are still developing, despite various efforts towards standardisation [3]. Much VA work has relied on physicians reviewing interview material and coming to a conclusion on cause of death, following a process closely analogous to clinical practice in which history, signs, and symptoms are used to construct a differential diagnosis. Recently, computer-based probabilistic models have become an important way of interpreting VA data, as an alternative to case-by-case physician interpretation [4]. These have the advantage of being faster, cheaper, and more internally consistent than physician review, but may lack some subtlety and nuance. Some comparisons between physician review and modelled findings have previously been made [4]–[6]. As well as characterising all-age, all-cause mortality, applications of verbal autopsy have included cause of death determination among particular groups such as women of reproductive age [7], and for assessing community interventions [8].

However, the outputs from probabilistic models have some technical differences from those typically generated by physicians, since the likelihood of a particular cause of death is also estimated quantitatively as part of the modelling. Several likely causes can be reported for a single case, and a case may remain partially or wholly indeterminate, particularly where the VA interview material is scanty. These characteristics might seem problematic from a clinical perspective that instinctively seeks a conclusive single main cause of death for each case (even though this is sometimes fudged by labelling two commonly coexisting causes as a single entity, for example the cause “HIV/AIDS and tuberculosis”). However, since VA is normally applied as a step towards community-based analyses of cause-specific mortality and public health implications, rather than as an endpoint whose primary concern is the individual case, the outcomes are essentially epidemiologically rather than clinically oriented. The proportions of deaths within a population attributable to a particular cause (cause-specific mortality fractions, CSMFs) are particularly important. Thus some uncertainty at the individual level, and possibly multiple causes per case, are not in themselves problematic, but need analytical approaches that make good sense of the data. The public health imperative to understand causes of death in terms of age and sex is also important, in order to understand burdens of premature mortality, to target potential interventions, and to inform health systems development.

In this paper we aimed to assess appropriate methods for analysing and interpreting VA interview data at the population level, using both probabilistically modelled and physician-interpreted results. An example dataset from the Agincourt health and sociodemographic surveillance site (HDSS) in South Africa, a member of the INDEPTH Network (http://www.indepth-network.org), is taken to illustrate the approaches used. Existing physician-interpreted findings from the same dataset are compared with modelled results in the sense of how they are derived and analysed, leading to some comparisons between the two approaches. However, the intention here is not to validate either approach; rather the emphasis is on interpretation and analysis processes which can lead effectively from data on deaths to public health imperatives.

## Methods

The Agincourt HDSS covers rural communities located in northeast South Africa, near the Mozambican border, and has monitored a contiguous population of around 70,000 since 1992. The background to this work is described more fully elsewhere [9], in a paper which analyses cause-specific mortality from 6,153 deaths that occurred between 1992 and 2005, on the basis of cause of death as determined by physician review. These physician reviews of VA interview material were each initially undertaken by two physicians independently. If they did not agree as to cause, a third physician arbitrated in order to reach a consensual cause of death. If consensus could not be reached, then no cause of death was recorded.

The same VA interview data were compiled into an input file for the InterVA v.3 probabilistic VA interpretation model (http://www.interva.net) and processed into cause of death data. The InterVA model is based on Bayesian calculations of probabilities that a particular death was due to particular causes, given a set of symptoms and circumstances associated with the death. This is achieved using a probability matrix which generically estimates probabilities of particular symptoms and circumstances of death, given particular causes. The model was developed using an expert panel and was deliberately designed to be generic and not context-dependent, and to produce relatively broad cause-of-death categories [10]. As previously described [5], the model expects an input of “high” or “low” to reflect the local prevalence of two specific causes which often vary by more than an order of magnitude between settings: HIV and malaria; here these were set to “high” and “low” respectively. These settings do not override the handling of individual cases, but are conceptually similar to a physician knowing that a particular disease is common or rare in the local population, irrespective of a particular patient presenting in a consultation.

Compiling the data input file for the InterVA model (which consists of yes/no answers for each case on around a hundred questions relating to the VA interview material) may take some days for a data manager, but processing the file into causes of death using the model then only takes a matter of minutes. This contrasts with thousands of hours of physician time, and a cost in the region of US$20,000, for reviewing a dataset of this size. The model is also totally internally consistent, meaning that rerunning data produces exactly the same output, and there is also therefore complete consistency at the individual case level over time, when considering a series of deaths that actually occurred over many years. With physician interpretation, it is unlikely that the same physicians can be available to undertake this work over an extended period, and in any case it is probable that their thinking and understanding would change over time. The model provides up to three likely causes of death for each case, or concludes that the cause is indeterminate. Each cause assigned is associated with a likelihood, and the sum of likelihoods of assigned causes has a maximum value of 1.00. If the sum of likelihoods of assigned causes is less than 1.00, then the difference reflects a lack of certainty about the overall case. It therefore seems logical to regard this uncertain proportion of each case as an indeterminate component.

For analysis, a dataset was constructed from the model's output (using Microsoft FoxPro) in which each case had one or more records, each record having one cause (including the possible cause “indeterminate”) and a weight corresponding to the likelihood of that cause for the particular case. Thus over the whole dataset, the sum of all the weights was equal to the number of cases, 6,153. This dataset included a total of 11,834 records, an average of 1.92 per case. This data structure also facilitates the import of other background factors of interest (since every record contains the individual identifier variable), which can then be analysed against particular causes of death in a weighted multivariate model. Physician-interpreted material where consensus on a single main cause is required can be analysed in very similar ways, with the conceptual weighting for each case being 1.

The analyses presented here were carried out using Stata 10.

Surveillance-based studies in the Agincourt subdistrict were reviewed and approved by the Committee for Research on Human Subjects (Medical) of the University of the Witwatersrand, Johannesburg, South Africa (protocol M960720). Informed consent was obtained at the individual and household levels at every follow-up visit, whereas community consent from civic and traditional leadership was secured at the start of surveillance and reaffirmed from time to time.

## Results

The same 6,153 deaths as presented previously using physician-interpreted causes [9] are shown in Table 1, with cause of death as determined from the same VA material by the InterVA model and shown by cause and age–sex group. The physician-determined CSMFs for the overall population are also shown for comparison. The ten highest ranking causes constituted 83.3% of the total according to physician interpretation and 88.2% according to probabilistic interpretation, and 8/10 of these causes were the same according to both approaches (HIV, tuberculosis, chronic cardiac, diarrhoea, pneumonia/sepsis, transport-related accidents, homicides, and indeterminate). The fractional causes of death from the model reflect the aggregation of likelihoods of particular causes over age–sex subgroups within the Agincourt population. These subgroups are the same as those used for the input file to the InterVA model.

**Table 1:** Verbal autopsy findings for 6,153 deaths in Agincourt HDSS occurring between 1992 and 2005, by likely cause and age–sex group, using cause of death as interpreted probabilistically by the InterVA 3 model.

Cause of Death	Physician CSMF* (%)	InterVA CSMF* (%)	Number of Deaths by Age–Sex Group from InterVA Results							
			Up to 28 Days	28 Days to 1 Year	1–4 Years	5–14 Years	Men, 15–49 Years	Women, 15–49 Years	50–64 Years	65+ Years
Accidental drowning	0.2	0.2	—	—	2.3	10.3	—	—	0.5	—
Accidental poisoning	0.3	0.5	—	—	8.7	2.8	16.1	—	3.6	0.7
Acute cardiac	0.3	0.3	—	—	—	—	3.2	1.0	6.2	6.7
Acute respiratory	1.1	0.6	1.8	5.0	1.8	0.4	4.9	2.2	6.9	15.5
Bloody diarrhoea	0.1	0.6	—	2.3	13.4	1.9	2.7	5.8	1.4	12.2
Chronic cardiac	4.2	4.4	0.6	—	—	2.3	28.1	21.4	86.8	134.4
Chronic respiratory	0.7	1.9	—	1.1	0.5	—	7.2	6.0	33.1	70.7
Congenital malformation	0.5	0.2	9.6	0.8	—	—	—	—	—	—
Diabetes	1.2	2.9	—	—	4.2	2.0	17.8	22.9	38.9	93.5
Disease of nervous system	0.9	0.0	—	—	—	—	0.8	—	0.3	—
HIV/AIDS related	18.6	15.3	1.6	129.4	201.7	12.8	174.2	349.6	67.4	6.1
Haemoglobinopathy	0.1	0.4	—	—	7.4	10.3	2.8	6.9	—	—
Homicide	2.7	2.9	—	—	—	0.5	121.2	21.8	25.2	11.4
Indeterminate	34.8	31.0	71.1	117.8	166.7	64.0	341.0	323.0	296.8	524.7
Kidney/urinary disease	0.6	1.5	—	—	1.8	1.3	8.8	3.2	19.6	56.4
Kwashiorkor	1.3	0.0	—	—	2.5	—	—	—	—	—
Liver disease	1.6	4.0	—	2.7	5.0	2.6	32.1	21.2	54.5	129.2
Malaria	1.6	0.1	—	—	0.1	1.4	2.2	4.2	0.7	—
Malignancy	5.0	0.9	—	—	—	—	4.8	11.5	10.0	29.7
Malnutrition	0.3	0.2	—	—	1.7	1.0	—	—	0.2	12.0
Maternity related	0.5	0.4	—	—	—	—	—	26.8	—	—
Measles	0.1	0.0	—	—	0.8	—	—	—	—	—
Meningitis	0.7	1.4	7.1	8.4	8.1	19.6	13.6	8.8	11.1	8.3
Nonbloody diarrhoea	3.4	2.2	15.1	82.3	26.4	4.4	1.3	7.0	4.9	1.5
Other digestive disease	0.9	0.1	—	—	—	—	—	—	4.9	1.5
Other fatal accident	1.8	0.2	—	0.9	0.5	0.9	1.5	0.8	0.4	7.2
Pneumonia and/or sepsis	2.3	3.7	30.7	47.6	23.1	7.9	12.2	9.0	28.7	69.7
Preterm or small baby	0.9	0.2	9.9	2.2	—	—	—	—	—	—
Stroke	4.4	0.7	—	—	—	—	2.2	4.3	11.7	25.9
Suicide	1.2	0.8	—	—	—	3.9	27.6	2.5	5.4	8.0
Tetanus	0.1	0.2	—	1.0	—	0.7	0.6	0.6	3.4	7.1
Transport-related accident	2.6	3.3	—	0.5	13.2	25.7	96.8	30.1	18.9	17.0
Tuberculosis (pulmonary)	5.3	18.5	0.7	5.0	17.3	12.5	369.6	250.4	165.2	318.4
**All causes**	**100**	**100**	**148**	**407**	**507**	**189**	**1,292**	**1,134**	**903**	**1,573**

Cause-specific mortality fractions from physician coding of the same dataset are shown for comparison in the second column.

*Cause specific mortality fraction, across all ages.

The overall proportion of indeterminate cases was 31.0%, compared with 34.8% in the physician review process. This indeterminate category included 359 deaths for which verbal autopsies were not successfully completed. In the InterVA model, a further 375 cases were rated as completely indeterminate, and the summed weights of the uncertain proportion of the remaining cases totalled 1,170.9, an average uncertainty per case of 24.3%. In the physician coding, 1,609 individual cases were considered to be indeterminate, either because of insufficient information or failure to reach a consensus between assessing physicians. The physicians considered a further 173 cases as indeterminate for particular reasons, for example sudden deaths of unknown cause. It was also interesting to note that the physician coding process led to using a total of 250 different ICD-10 codes, but the ten most frequently used ICD-10 codes accounted for 70.7% of the deaths.

Table 2 shows the five principal causes of death for each age group and period. It has been constructed to be as similar as possible to the corresponding table in the previous paper using physician interpretation of the same dataset (Table 2 in [9]), a process which involved regrouping InterVA causes of death accordingly. For each period and age group, the physician-interpreted ranks from the previous paper are also shown for comparison.

**Table 2:** Five main causes of death by age group and time period, based on 6,153 deaths in Agincourt HDSS occurring between 1992 and 2005, using cause of death as interpreted probabilistically by the InterVA 3 model.

Age Group	Rank	1992–1994			1995–1997			1998–2001			2002–2005		
		Cause	%	PR*	Cause	%	PR*	Cause	%	PR*	Cause	%	PR*
**0–4 Years**	Indeterminate		43.8			38.6			35.7			25.6	
	1	Diarrhoea	24.7	1	HIV/TB	20.8	2	HIV/TB	31.0	1	HIV/TB	49.7	1
	2	HIV/TB	9.7	—	Diarrhoea	20.5	1	Acute resp infection	11.5	3	Acute resp infection	9.9	3
	3	Acute respiratory infection	9.2	—	Acute respiratory infection	10.0	3	Diarrhoea	11.2	2	Diarrhoea	7.5	2
	4	Other infection	3.4	5	Other NCD	3.3	—	Other infection	2.3	—	Other infection	2.3	—
	5	Accidental injury	3.3	4	Other infection	2.8	—	Perinatal causes	1.7	4	Congenital	1.2	—
**5–14 Years**	Indeterminate		33.4			23.6			34.7			38.2	
	1	Other infection	17.5	—	Accidental injury	12.3	3	Other infection	15.8	—	HIV/TB	20.1	2
	2	Road traffic accident	12.8	2	Other infection	11.5	—	Road traffic accident	14.3	5	Road traffic accident	15.8	3
	3	HIV/TB	11.4	—	HIV/TB	10.6	4	Accidental injury	8.4	1	Accidental injury	7.5	1
	4	Other NCD	8.0	1	Other NCD	8.6	2	HIV/TB	6.9	3	Other infection	5.7	5
	5	Diarrhoea	5.9	—	Road traffic accident	8.5	5	Other NCD	5.5	—	Other NCD	5.7	4
**15–49 Years**	Indeterminate		30.3			31.1			27.3			26.3	
	1	HIV/TB	23.8	3	HIV/TB	34.3	1	HIV/TB	44.2	1	HIV/TB	54.4	1
	2	Assault	14.3	1	Assault	9.6	2	Road traffic accident	6.7	3	Assault	4.4	2
	3	Road traffic accident	12.6	2	Road traffic accident	7.7	3	Assault	5.1	2	Road traffic accident	2.9	3
	4	Maternity	3.3	—	Chronic liver	2.8	—	Other NCD	3.3	4	Other NCD	2.2	5
	5	Other NCD	3.0	—	Other NCD	2.8	4	Chronic liver	2.6	—	Chronic liver	2.0	—
**50–64 Years**	Indeterminate		36.6			38.0			32.2			30.6	
	1	HIV/TB	14.0	—	HIV/TB	21.5	1	HIV/TB	22.0	1	HIV/TB	31.9	1
	2	Other NCD	11.3	4	Other cardiac	12.6	3	Other cardiac	9.4	—	Other cardiac	9.4	3
	3	Other infection	7.8	—	Other NCD	10.0	4	Chronic liver	9.3	—	Other NCD	7.9	4
	4	Other cardiac	6.7	3	Chronic liver	4.2	—	Other NCD	7.6	3	Chronic liver	5.0	—
	5	Chronic liver	6.2	2	Assault	4.1	5	Other infection	4.3	—	Acute respiratory infection	3.9	—
**65+ Years**	Indeterminate		37.4			34.1			31.6			32.9	
	1	Tuberculosis	22.4	3	Tuberculosis	21.1	3	Tuberculosis	20.7	4	Tuberculosis	18.4	4
	2	Other cardiac	7.4	1	Other cardiac	10.5	2	Other NCD	13.5	5	Other NCD	11.3	5
	3	Acute respiratory infection	7.1	—	Other NCD	7.4	—	Chronic liver	9.7	—	Other cardiac	9.0	2
	4	Other NCD	6.8	—	Acute respiratory infection	6.9	—	Other cardiac	7.3	3	Chronic liver	8.9	—
	5	Chronic liver	6.4	5	Chronic liver	6.0	—	Acute respiratory infection	5.0	—	Other infection	5.8	—

*PR are physician-interpreted ranks within each period and age category, as previously published [9].

NCD, noncommunicable disease.

One instance in which there was a clear difference in the estimates between the physician-coded and modelled findings was in tuberculosis as a cause of death among the elderly (over 65 years). According to the physicians, 96/1,492 (6.4%) of deaths in this age group were due to tuberculosis, compared with 318.4/1,492 (21.3%) according to the model. Of the 96 cases reported as tuberculosis by the physicians, 78 (81.3%) were also concluded to be tuberculosis by the model. However, among the 241 cases rated as tuberculosis by the model but not by the physicians, 103 (42.7%) were rated as indeterminate by the physicians. To elucidate this difference, Table 3 shows the breakdown of key VA interview parameters which might contribute to a conclusion of tuberculosis as cause of death, both for the InterVA model and for the physicians. It includes the positive predictive value (PPV) for tuberculosis for each parameter, both in the physician and model interpretation.

**Table 3:** Pulmonary tuberculosis as a possible cause of death among 1,492 elders (65+ years) as interpreted by physician consensus (6.4%) and probabilistic modelling (21.3%), in relation to selected verbal autopsy parameters.

VA Parameter		Physician Interpretation			Probabilistic Modelling		
		Not TB	TB	PPV*	Not TB	TB	PPV*
Chest pain	No	1,041	8	91%	949	100	69%
	Yes	359	84		225	218	
Chronic cough	No	1,155	6	93%	1,067	94	70%
	Yes	245	86		107	224	
Productive cough	No	1,179	8	91%	1,059	128	60%
	Yes	221	84		115	190	
Difficulty breathing	No	794	29	68%	687	136	58%
	Yes	606	63		486	183	
Night sweats	No	1,274	41	55%	1,143	172	46%
	Yes	126	51		31	146	
Chronic fever	No	908	47	49%	846	109	66%
	Yes	492	45		328	209	
Weight loss	No	426	5	95%	414	26	92%
	Yes	974	87		769	292	
History of tuberculosis	No	1,352	40	57%	1,168	225	26%
	Yes	48	52		18	82	

*PPV: positive predictive value of the VA parameter for pulmonary tuberculosis.

## Discussion

Having considered the causes of more than 6,000 deaths over a 14-year period, the ten highest-ranking causes accounted for 83% and 88% of all deaths by physician interpretation and probabilistic modelling respectively, and eight of the highest ten causes were common to both approaches. Probabilistic modelling was cheaper and more internally consistent than physician interpretation. Uncertainty around the cause(s) of individual deaths was recognised as an important concept that should be reflected in any overall analysis of cause-specific mortality.

The advantages and disadvantages of physician-interpreted and probabilistically modelled cause of death data as evidenced by these analyses were largely as anticipated. Physician-interpreted findings included a number of quite specific, but rare, causes which were not designed to be addressed by the current model. While it is possible to build similar models with more detailed inputs and outputs, as has been done for deaths among women of reproductive age [7], this model was designed to capture major cause-of-death groupings. In principle a model designed to include greater differentiation—for example, between different cancers at particular sites—could be constructed; but the extent to which that would lead to greater understanding of population health is less clear. The very large number of specific causes used by the physicians, even though the occurrence of many was very low, could be regarded as an advantage in terms of subtlety or as a disadvantage in terms of clear overall understanding of mortality patterns (without applying further judgement calls on appropriate grouping). Probabilistically-modelled interpretation has major advantages in terms of cost (not needing to pay physicians), time (less delay in getting results after interviews) and complete consistency. A recent review of the Indepth Network accordingly concluded that the InterVA model represented the most effective way forward for standardised interpretation of VA data across the network. [11] However, there is also the possibility of there being consistent errors encapsulated in the model.

The methods described here for analysing the probabilistically modelled cause of death data are relatively straightforward, taking into account that particular causes of death have been modelled with a specific likelihood and the quantifiable margin of uncertainty associated with many individual cases. These methods allow the margins of uncertainty associated with individual cause of death assignments to be carried through into the aggregated analysis process.

When physicians are used to assess VA material, and particularly if, as was the case for these data, physician consensus on individual cases is taken to be an important part of the process [12], then a simpler analytical approach can be used, as evidenced in the earlier paper using these data [9]. Once each case is assigned a cause of death or is considered to be indeterminate, categorising and tabulating cases as needed is straightforward, since each death counts as a single case. However, it has to be realised in this approach that any sense of the uncertainty that may have been evident in the original physicians' consideration of individual cases, or in consensus conferences, has already been eliminated before aggregated analysis begins. Since both approaches yielded only about two-thirds certainty, incorporating uncertainty in aggregated measures of cause-specific mortality seems important. Uncertainty might be better handled in physician-interpreted data if individual physicians' opinions were used, rather than insisting on consensus.

Given the very different approaches to cause-of-death interpretation and analysis as presented here and in the earlier paper (probabilistic modelling and analysis incorporating uncertainty, versus physician assessment and tabulation of definitive consensus findings), it is perhaps remarkable that many of the salient features of Table 2 here and Table 2 in the previous paper are closely similar [9]. Both give a picture of a population increasingly dominated by the burden of HIV-related mortality as time passes, together with appreciable numbers of deaths due to external causes, and relatively low infectious disease mortality (apart from the HIV/TB combination). It is also interesting to note that the overall proportion of cases to which specific causes could not be attributed is similar, despite being derived from completely different methods.

There are also some potentially important differences emerging from the two approaches, even though they are not huge in the context of the entire dataset. In considering any such differences, it has to be recognised that there is no gold standard available here. Kahn et al. have previously undertaken a validation exercise between physician assessments and a limited number of well-justified hospital-based diagnoses [12], and we plan to extend this to a detailed three-way comparison including the InterVA findings for this limited subset of deaths. However, in a community such as this where many people die without contacting health services, and where hospital records are often of poor quality, the quest for a wide-ranging gold standard for VA findings which fairly represents all causes and circumstances of death has to be regarded as futile.

Notable differences that do emerge include lower estimates of malignant disease in the InterVA findings and lower estimates of tuberculosis among the elderly in the physician data. The InterVA model also gave higher estimates of HIV-related mortality in the first period (1992–94), which is particularly interesting to note. This early difference may reflect a degree of false-positive HIV-related findings by the model during a period of lower HIV-prevalence, and this needs to be further investigated in terms of characterising the overall HIV prevalence for the model as “high”. On the other hand, it might reflect a difficulty among the physicians in achieving consensus on HIV as a cause of death in those relatively early days of the epidemic, and this is also something to look into further. It seems likely that during the onset of the epidemic, individual physicians' perceptions of new disease patterns might have developed quite rapidly, but not necessarily in the same ways and at the same rates, depending on their personal experiences. This process, at least for a while, might have increased the difficulties in achieving consensus on HIV-related causes. The reported rate of ill-defined or unknown causes was highest in the physician-coded material for the period 1992–94 (approximately one-third), falling to approximately one-fifth by 2002–04 [9].

The examples of factors leading to tuberculosis as a cause of death among the elderly, as detailed in Table 3, provide interesting insights into differences between the two interpretations. It is clear that the physicians mainly determined tuberculosis as a cause when chest pain, chronic cough, productive cough, and weight loss were all reported for a particular case, whereas the model took a less specific approach. This is reflected in the generally higher positive predictive values for physician interpretation. On the other hand, the high proportion of indeterminate conclusions reached by the physicians among the model's probable tuberculosis cases suggests a degree of uncertainty in their deliberations, rather than clear alternative conclusions. There may also be a question of physicians' expectations of the likelihood of tuberculosis among the elderly, given that many elders in this community will now be living in households with younger adults coinfected with HIV and tuberculosis. Recent studies from Spain [13] and China [14] reported raised tuberculosis case-fatality rates among the elderly. In any case, although this example represented one of the larger discrepancies between the two approaches, it still accounted for only 218/6,153 (3.5%) of overall deaths.

The importance of conceptual categorisations of cause of death can also be seen in these comparisons. At first sight, it appears that the approaches (Table 2) gave different pictures regarding deaths due to malnutrition among the under-5s, with 0.4% from the model and 9.0% from the physicians [9]. However, if one considers that tuberculosis is probably a relatively rare cause of death in young children, even as an HIV coinfection (as evidenced in nearby Mozambique [15]), and that HIV-infected children are more likely to follow a pattern of chronic diarrhoea and malnutrition [16], then the picture changes somewhat. So, taking the physicians' “HIV/tuberculosis” grouping as mainly not being tuberculosis in this age group, and adding that to their “diarrhoea” and “malnutrition” codings, for the under-5s the proportions of deaths due to “HIV/diarrhoea/malnutrition” were 38%, 41%, 42%, and 52% for the four periods, respectively. This result is strikingly similar, in magnitude and progression, to the same grouping from the InterVA findings (34%, 41%, 42%, and 56% respectively), and would represent the largest single cause of under-5 mortality in both approaches. Thus conceptual groupings that reflect real public health issues, rather than (in this instance) rather sterile debates as to what HIV-infected children with chronic diarrhoea and wasting actually die from, are crucial. International Classification of Diseases (ICD-10) coding for causes of death may not therefore be as relevant at this conceptual level, even if they can be a useful framework at earlier stages, for example in assigning physician-coded causes.

The main aim of this paper is not to provide a validation of any particular VA method, but to consider alternative approaches for handling interview data on individual deaths to give meaningful pictures of population health. These data are the basic resource for public health planning: the questions in our minds throughout these considerations have started from *“If I were the local Director of Public Health…”*. From these data, and irrespective of the methods used for analysis and interpretation, it is clear that the Agincourt population has undergone rapid changes, which imply new intervention target groups, expanded demands on health professionals' skills, changing demands on health services and increasing resource requirements. The pictures of the major public health themes within the Agincourt population that emerge from both of the interpretative approaches considered are encouragingly similar, both in terms of overall cause-specific mortality patterns and in the ways that they have tracked changes over time, and the adoption of one or other method of interpretation would not lead to fundamentally different public health actions. The clear development of the HIV epidemic revealed in this example, and seeing which population subgroups are vulnerable to particular diseases, both highlight some of the advantages of using VA as a public health tool. At least where VA is used within routine health services, probabilistic modelling with its consistent approach over time and place, the elimination of inter- and intra-assessor variation, faster results, and much lower cost, should be the interpretative method of choice.

## Abbreviations

CSMF: cause-specific mortality fraction
HDSS: health and sociodemographic surveillance site
PPV: positive predictive value
VA: verbal autopsy
